# Temperature independence of piezoelectric properties for high-performance BiFeO_3_–BaTiO_3_ lead-free piezoelectric ceramics up to 300 °C†

**DOI:** 10.1039/c8ra07553k

**Published:** 2018-10-19

**Authors:** Li-Feng Zhu, Qing Liu, Bo-Ping Zhang, Zhen-Yong Cen, Ke Wang, Jun-jie Li, Yang Bai, Xiao-Hui Wang, Jing-Feng Li

**Affiliations:** School of Materials Science and Engineering, University of Science and Technology Beijing Beijing 100083 China bpzhang@ustb.edu.cn; State Key Laboratory of New Ceramics and Fine Processing, School of Materials Science and Engineering, Tsinghua University Beijing 100084 China wang-ke@tsinghua.edu.cn; Key Laboratory of Environmental Fracture (Ministry of Education), University of Science and Technology Beijing Beijing 100083 China

## Abstract

The temperature-dependence behaviors of ferroelectric, piezoelectric, *k*_p_ and electrical-field-induced strain were carefully evaluated for high-performance BiFeO_3_–0.3BaTiO_3_ (BF–0.3BT) ceramics. There results indicate, combined with Rayleigh analysis and temperature-dependence XRD and PFM, that the increase of strain and large signal 
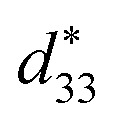
 with increasing the temperature from room temperature to 180 °C is related to the joint effect of intrinsic contribution (lattice expansion) and extrinsic contribution (domain switching). With further increasing the temperature to 300 °C, the large signal *d*_33_ and electrical-field-induced strain mildly decrease because of the increase of conductivity for BF–0.3BT ceramics. However, different from strain and large signal 
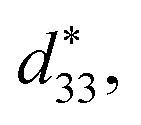
 the small signal *d*_33_(E_0_) and *k*_p_ exhibit excellent temperature stability behavior as the temperature increases from room temperature to 300 °C.

## Abstract 2

The temperature-dependence behaviors of ferroelectric, piezoelectric, *k*_p_ and electrical-field-induced strain were carefully evaluated for high-performance BiFeO_3_–0.3BaTiO_3_ (BF–0.3BT) ceramics.

## Graphical abstract

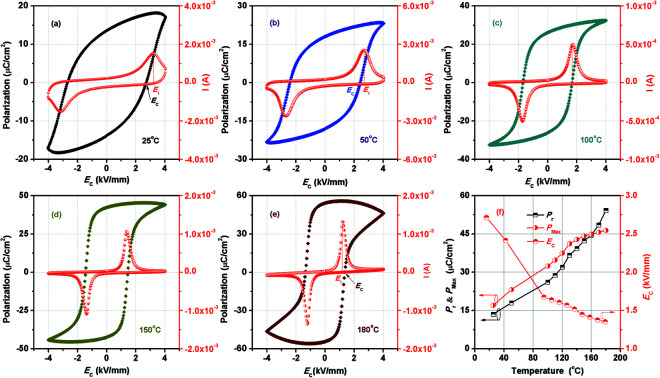

## 1. Introduction

Piezoelectric materials can realize the interconversion of mechanical energy and electrical energy and have been widely used in actuators, transducers and sensors, and so on.^1^ With the enhancement of the awareness of environmental protection, the Pb(Zr,Ti)O_3_ (PZT) family, being the most dominant piezoelectric materials, is faced with global restrictions because of the considerable portion of hazardous lead it contains. Therefore, it is urgent to seek lead-free alternative materials which have comparable electromechanical properties to these lead-based counterparts.^2–20^ A series of typical lead-free piezoelectric ceramics, such as: BaTiO_3_,^5–7^, (1 − *x*)Bi_0.5_(Na,K)_0.5_TiO_3_–*x*BaTiO_3_,^8,9^, (K_1−*x*_Na_*x*_)NbO_3_ (ref. 10–14) and BiFeO_3_–BaTiO_3_ (BF–BT),^15–20^ have been extensively investigated in recent years. Some of them exhibit excellent piezoelectric properties, such as: *d*_33_ = 620 pC N^−1^ in the Ba_0.7_Ca_0.3_TiO_3_–50BaZr_0.2_Ti_0.8_O_3_ system^6^ and *d*_33_ = 570 pC N^−1^ in the K_1−*w*_Na_*w*_Nb_1−*z*_Sb_*z*_O_3_–*y*BaZrO_3_–*x*Bi_0.5_K_0.5_HfO_3_ system,^12^ in which the *d*_33_ is comparable to that of the PZT system. However, these lead-free ceramics do not meet the industrial application requirements because of their inferior temperature-insensitive behavior and low Curie temperature, such as: *T*_C_ = 93 °C in the Ba_0.7_Ca_0.3_TiO_3_–50BaZr_0.2_Ti_0.8_O_3_ system^6^ and ∼250 °C in the K_1−*w*_Na_*w*_Nb_1−*z*_Sb_*z*_O_3_–*y*BaZrO_3_–*x*Bi_0.5_K_0.5_HfO_3_ system.^12^ Thus, the task of substituting for PZT ceramics fell on the BF–BT lead-free piezoelectric system because it has a high *T*_C_, over 580 °C,^16^ and a morphotropic phase boundary (MPB), which is similar to PZT system. Nevertheless, comparing with the PZT system, the electrical properties of BF–BT ceramics, especially the piezoelectric property, resistance and dielectric loss, need to be improved.

Recently, considerable efforts have been carried out to address this problem mentioned above ,^16–31^ such as: (1) adding oxide additives, *e.g.*, MnO_2_,^16,20,21^ Bi_2_O_3_,^18,22^ CuO,^23^ La_2_O_3_,^24^ and so on, have been added to improve the resistivity as well as the piezoelectric properties. (2) Building ternary system, *e.g.*, BF–BT–Bi(Mg_1/2_Ti_1/2_)O_3_,^19^ BF–BT–Bi(Zn_1/2_Ti_1/2_)O_3_,^17,25,26^ BF–BT–Bi_0.5_K_0.5_TiO_3_,^27^ and so on. (3) Ion substitutions. Ion substitutions for Bi^3+^ (*e.g.*, La^3+^, Er^3+^)^28^ or Fe^3+^ (*e.g.*, Ga^3+^, Al^3+^, Sc^3+^, Co^3+^)^20,29^ in BF–BT system were carried out to improve the electrical properties. It is worth mentioning that the BF33BT–3BG ceramics, prepared by the water-quenched process, reported by Lee^20^ exhibit excellent piezoelectric properties (*d*_33_ = 402 pC N^−1^) and high Curie temperature (*T*_C_ = 454 °C), indicating that the BF–BT system is the most promising candidate to replace Pb-based piezoelectric ceramics.

It is well known that industrial implementation not only demands good piezoelectric performance at room temperature but also hopes an excellent stability for piezoelectric properties in response to temperature changes. Despite the *d*_33_ of BF–BT system has achieved major breakthroughs^20^ and even it can meet the industrial application, how about the temperature stability of piezoelectric properties for the BF–BT ceramics is, and whether it is similar with the PZT system? Very few articles^31^ point out that the ferroelectric property for BF–BT ceramics was enhanced with increasing the temperature from room temperature to 150 °C. Nevertheless, there have been few investigations of the temperature dependence of piezoelectric properties for BF–BT ceramics. In addition, the reasons which cause the improvement of ferroelectric and *k*_p_ properties are still vague for BF–BT ceramics so far.

In this work, 0.7BiFeO_3_–0.3BaTiO_3_ (abbreviated as BF–0.3BT) ceramics were prepared to reveal the temperature-dependence behavior of its ferroelectric, piezoelectric, *k*_p_ and electrical-field-induced strain properties in the wide temperature range between room temperature and 300 °C. Because of the joint effect of intrinsic contribution (lattice expansion tested by temperature-dependence XRD) and extrinsic contribution (domain switching proved by temperature-dependence PFM), the remanent polarization (*P*_r_), strain and large signal 
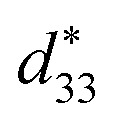
 increase with increasing the temperature from room temperature to 180 °C. This result is also verified by Rayleigh analysis models, which indicates that the extrinsic contribution and intrinsic contribution (*d*_int_ × 10^−12^) increase from 18.70% and 161.84 m V^−1^ at 24 °C to 34.45% and 207.06 m V^−1^ at 100 °C. With further increasing the temperature from 180 °C to 300 °C, the large signal *d*_33_ and electrical-field-induced strain mildly decrease. However, different the strain and large signal 
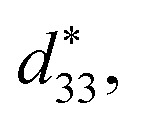
 the small signal *d*_33_(E_0_) and *k*_p_ for BF–0.3BT lead-free piezoelectric ceramics exhibit an excellent temperature-insensitive behavior at measured temperature range between the room temperature and 300 °C because it has high *T*_C_ ∼475 °C and stability phase structure below *T*_C_, which is even superior to PZT system. This result offers an evidence for industrial application of BF–BT ceramics to replace Pb-based piezoelectric ceramics in high-temperature piezoelectric devices.

## 2. Experimental procedure

Nano-BaTiO_3_ (about 100 nm, 99%), Fe_2_O_3_ (99%) and Bi_2_O_3_ (99%) powders were used as raw materials, which were weighed on the basis of a composition of 0.7BiFeO_3_–0.3BaTiO_3_ (abbreviated as BF–0.3BT), and mixed in nylon tank. After dried and calcined at 800 °C for 12 h, the resultant powders were milled again and pressed into disks of 10 mm in diameter and 1.5 mm in thickness under 80 MPa using 3 wt% polyvinyl alcohol (PVA) as the binder, followed by burning the binder at 650 °C by 5 °C min^−1^ and for 2 h. After removing the PVA, the samples were sintered at 1000 °C with holding for 6 h, and then furnace cooled to room temperature. The sintered specimens were coated with silver for the electrical measurement. The coated samples were poled under a DC field of 4 kV mm^−1^ at 130 °C for 30 min in a silicone oil bath.

The crystallographic structures were investigated by using X-ray diffraction (XRD: D/max-RB, Rigaku Inc., Japan) with a Cu Kα radiation (*λ* = 0.15406 nm) filtered through a Ni foil. Bulk densities of samples were measured using the Archimedes method. The temperature dependence of dielectric properties was examined using a programmable furnace with an LCR analyzer (TH2828S) at 1 kHz in temperature range of 20 °C to 650 °C. Microstructure of the sintered samples was observed by field emission scanning electron microscopy (FESEM, SUPRATM 55, Japan). Piezoelectric properties were measured using a quasi-static piezoelectric coefficient testing meter (ZJ-3A, Institute of Acoustics, Chinese Academy of Sciences, Beijing, China). Field-dependent and temperature-dependent parameters including *d*_33_(*E*) hysteresis loops, ferroelectric hysteresis (*P*–*E*) loops and unipolar strain *S*(*E*) were measured with ferroelectric measuring equipment (aixACCT TF Analyzer 2000, Germany). The temperature-dependent planar electromechanical coupling coefficient *k*_p_ was determined by resonance-antiresonance method using an Agilent 4294A precision impedance analyzer (Hewlett-Packard, Palo Alto, CA). The temperature-dependent of leakage currents (*J*) were measured by precision 10 kV HVI-SC precision materials analyzer (Radiant technologies INC, USA). In addition, the PFM experiments were carried out by a commercial atomic force microscope (MFP-3D, Asylum Research, USA)

## 3. Results and discussion

**Fig. 1:**
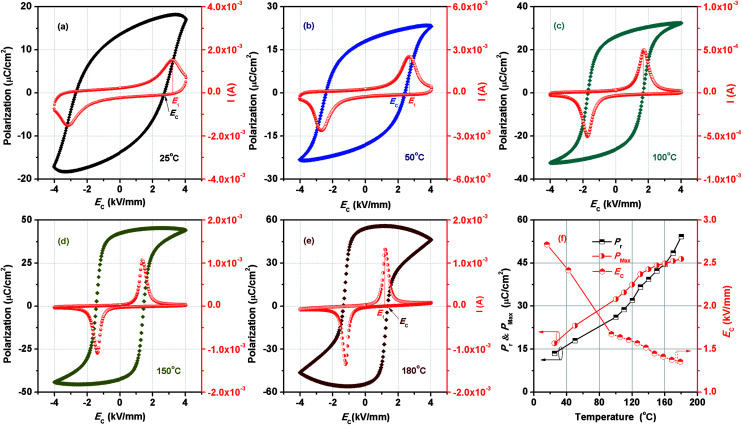
Temperature dependence of *P*–*E* loops of BF–0.3BT ceramics (a–e), and the variation of *P*_r_, *P*_max_ and *E*_C_ with the different temperature (f).

Temperature-dependent *P*–*E* loops of BF–0.3BT ceramics under a field of 4.0 kV mm^−1^ at the frequency of 1 Hz are shown in Fig. 1(a–e). The samples exhibit a relative saturation *P*–*E* hysteresis loops measured at room temperature as shown in Fig. 1(a) and only a single polarization current peak located in *E*_1_ corresponding to the normal domain switching is observed during electric loading. However, the *E*_1_ is larger than coercive field (*E*_C_). This indicates that the domain switching is suppressed by the defect dipole under a low electric field. With increasing the temperature to 100 °C, a typical ferroelectric polarization hysteresis loop is detected, along with *E*_1_ = *E*_C_, indicating that increase of temperature is benefit for reducing the pinning of defect dipole and promoting the domain switching. As the temperature reaches to 180 °C, the *E*_1_ is lower than *E*_C_ as shown in Fig. 1(e), suggesting that the conductivity of samples increases, caused by the increase of migration of defect ions. The detailed variations of *P*_r_, *P*_max_ and *E*_C_ with different temperature are shown in Fig. 1(f). As the temperature increases, the *E*_C_ monotonously decreases. Instead, the *P*_r_ and *P*_max_ from 13.57 μC cm^−2^ and 17.03 μC cm^−2^ increase to 54.23 μC cm^−2^ and 46.44 μC cm^−2^ with increasing the temperature from 25 °C to 180 °C, respectively. In addition, it also can find that the *P*_r_ is larger than *P*_max_ when the temperature is over 160 °C. This may be because the fact that the leakage abruptly increases as the temperature is over 160 °C as shown in Fig. S1.†

**Fig. 2:**
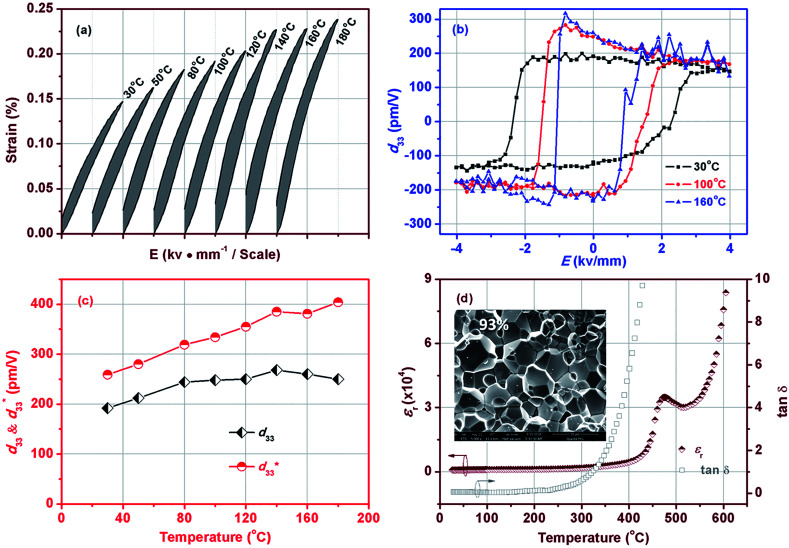
Temperature dependence of unipolar strain of BF–0.3BT ceramics at 5.5 kV mm^−1^ field excursion (a), temperature dependence of *d*_33_–*E* loops (b), *d*_33_ and 
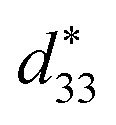
 values (c), temperature dependent dielectric constant *ε*_r_ and dielectric loss factor tan *δ* (d) for BF–0.3BT ceramics.

Temperature-dependent electrical-field-induced strain (*S*–*E* loop) under a field of 5.5 kV mm^−1^ is shown in Fig. 2(a). The strain of samples measured at room temperature is 0.15%, which is comparable to that of soft PZT ceramics (typical strain values 0.15% at 2 kV mm^−1^)^32^ and 0.95(Na_0.49_K_0.49_Li_0.02_) (Nb_0.8_Ta_0.2_)O_3_–0.05CaZrO_3_ (CZ5) ceramics (0.16% at 6 kV mm^−1^).^13^ With increasing the temperature, the strain increases. This phenomenon is consistent with the PZT system, such as PZT-5H^32^ and PZT4,^10^ indicating that the BF–0.3BT ceramics exhibit slight temperature-dependent behaviors. As the temperature increases from 30 °C to 180 °C, the strain increases from 0.15% to 0.237%. Different from strain, the small signal *d*_33_(*E*) exhibits few changes with increasing the temperature as shown in Fig. 2(b) and (c). Fig. 2(b) shows the field-dependent piezoelectric coefficient *d*_33_(*E*) loops, which were measured by applying a triangular signal of 4.0 kV mm^−1^ at the frequency of 1 Hz with an superimposed AC voltage of 25 V at 250 Hz, and were used to characterize the quasi-static *d*_33_. The equivalence between the piezoelectric constant value achieved by the *d*_33_(*E*) measurement and that acquired from quasi-static *d*_33_ meter has also been confirmed by Fialka *et al.*^33^ and Yao.^34^ Generally, the positive value at zero field in *d*_33_(*E*) loops is considered as piezoelectric constant *d*_33_. The small signal *d*_33_(*E*_0_) of BF–0.3BT ceramics is about 195 pm V^−1^ at room temperature that is comparable to the piezoelectric coefficient *d*_33_(205 pC N^−1^) measured by a quasi-static piezoelectric constant apparatus. Therefore, the temperature-dependence electric-field-dependent piezoelectric coefficient *d*_33_(*E*) loops were used to represent the temperature-dependence *d*_33_. From the Fig. 2(b), it can find that the small signal *d*_33_(*E*_0_) has little change as the temperature increases from 30 °C to 160 °C. The detailed variations for small signal *d*_33_(*E*_0_) and larger signal 
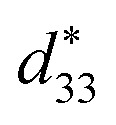
 with different temperature are shown in Fig. 2(c). With increasing the temperature from 30 °C to 180 °C, the 
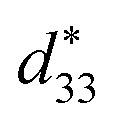
 increases from 260 pm V^−1^ to 404 pm V^−1^. It is well known that the piezoelectric coefficient is comprised of both an intrinsic 
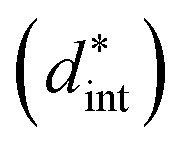
 and extrinsic 
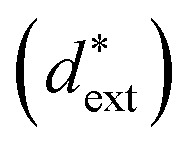
 components as shown in eqn (1). The intrinsic piezoelectric contribution is related to the lattice variation. However, the extrinsic contribution is associated with domain switching and phase boundary motions. Here the increases of 
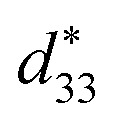
 with increasing temperature may also be ascribed to crystal lattice variation and domain switching. Different from large signal 
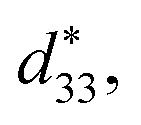
 small signal *d*_33_(*E*_0_) exhibits an evident temperature-stability behavior. This indicates that BF–0.3BT ceramics have a good promising in high temperature sensors areas. Fig. 2(d) shows the temperature dependence of dielectric permittivity *ε*_*r*_ and dielectric loss factor tan *δ* for BF–0.3BT ceramics measured at 1 kHz between 20 °C and 650 °C. Only one *ε*_*r*_ peak is detected, corresponding to Curie temperature *T*_C_ = 475 °C, which is superior to other lead-free piezoelectric systems, such as: KNN, BNT–BT, BT, and most Pb-based systems, further indicating that BF–BT ceramics have a good promising in high temperature sensors and actuators. In addition, the fracture surface SEM of samples is shown in inset of Fig. 2(d), in which few pores, caused by Bi_2_O_3_'s volatilization at sintering process, are detected. The relative density of the BF–0.3BT ceramics is about 93%.1
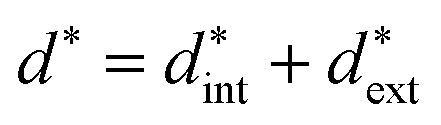


**Fig. 3:**
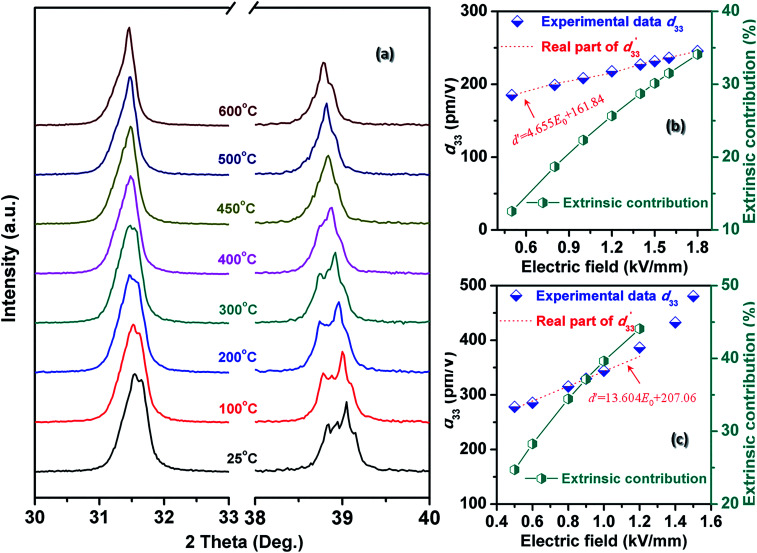
Temperature dependence of X-ray diffraction patterns for BF–0.3BT ceramics in a selected 2*θ* range of 30–33° and 38–40° (a), real part of 
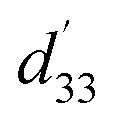
 and experimental *d*_33_, as well as extrinsic contribution ratio for BF–0.3BT ceramics measured at room temperature (b) and 100 °C (c).

In order to clarify the effect of intrinsic and extrinsic contribution on the *P*_r_, *d*_33_(*E*_0_) and 
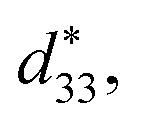
 the temperature dependence of XRD for BF–0.3BT ceramics was measured and shown in Fig. 3(a) and S2.† The crystal structure is *R* and *T* two-phase coexistence at room temperature as shown in Fig. S3† and does not change until the temperature over 450 °C as shown in Fig. 3(a) and S2.† Moreover, with increasing the temperature, the diffraction peaks shift to the low angle, indicating that the lattice expands as shown in Fig. 3(a). From XRD results, it is still difficult to exclude the effect of extrinsic contribution on the piezoelectric properties. The Rayleigh model, which was originally used for analyzing the nonlinear response of ferromagnetic materials^35,36^ and also successfully applied to ferroelectric materials to describe the linear dependence of piezoelectric constant on the electric field amplitude recently, is one of the simplest approaches to obtaining details on nonlinear phenomena due to the domain motion. Thus, the intrinsic and extrinsic contribution on strain and piezoelectric coefficient can be evaluated by using the Rayleigh model analysis as shown in Fig. S4.† The BF–0.3BT ceramics exhibit linear Rayleigh behavior over the range of 0.5 ≤ *E*_0_ ≤ 1.8 kV mm^−1^ measured at room temperature as shown in Fig. 3(b) and 0.5 ≤ *E*_0_ ≤ 1.0 kV mm^−1^ measured at 100 °C as shown in Fig. 3(c), respectively. With increasing the electric field, the extrinsic contribution linearly increases, resulting in the increase of *d*_33_. Comparing with the samples measured at room temperature, with increasing the temperature both of intrinsic and extrinsic contribution increase as shown in Table 1. The intrinsic contribution value is 161.84 pm/V at room temperature, and increases to 207.06 pm V^−1^ at 100 °C. This may be related to the lattice expansion. The ratio of extrinsic contribution using *E*_0_ = 0.8 kV mm^−1^ increases from 18.7% at 24 °C to 34.45% at 100 °C as shown in Table 1, indicating that the increase of temperature is propitious to domain switching.

**Table 1:** Rayleigh coefficient for BF–0.3BT ceramics with different temperature^a^

Temperature (°C)	24	50	80	100
*a*_d_ × 10^−17^ (m^2^ V^−2^)	4.655	8.624	11.282	13.604
*d*_int_ × 10^−12^ (m V^−1^)	161.84	177.93	193.84	207.06
Extrinsic contribution (%)	18.70%	27.94%	31.77%	34.45%
*R* ^2^	0.9952	0.9945	0.9967	0.9939

a The extrinsic contribution is estimated by eqn (S5 see ESI) using *E*_0_ = 0.8 kV mm^−1^.

**Fig. 4:**
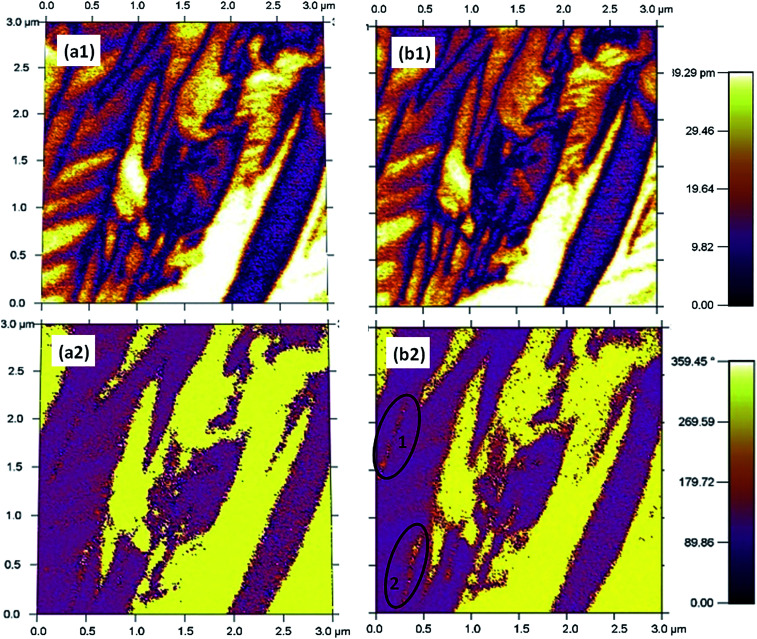
PFM scanning results of out-of-plane amplitude and phase for the sample measured at 25 °C (a1) and (a2), and 100 °C (b1) and (b2).

The apparatus PFM facilitates the observation of the components of spontaneous polarization vectors in domains, which consequently may shed light on the variation of the domain. *In situ* PFM phase and piezoresponse of BF–0.3BT ceramics were investigated at different temperatures 25 °C and 100 °C, as depicted in Fig. 4. Both micro and nano domain are observed in the samples at 25 °C. As the temperature increases to 100 °C, obvious domain switching (at 1 and 2 areas) was detected as shown in Fig. 4(b2), further verifying that the increase of temperature can release the pinning of ionic defect and facilitate the domain switching.

**Fig. 5:**
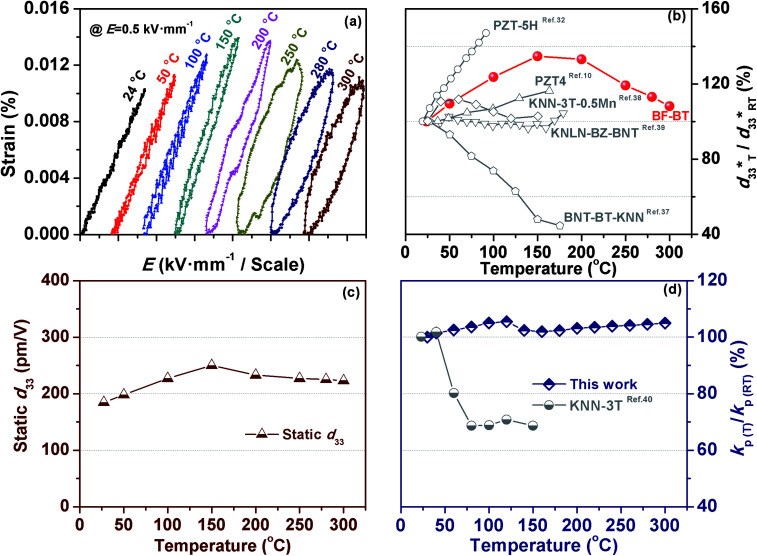
Temperature dependence of unipolar strain of BF–0.3BT ceramics at 0.5 kV mm^−1^ field excursion (a), comparison of temperature dependence of normalized strain 
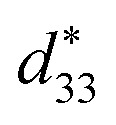
 for various piezoceramics as normalized to its room temperature value 
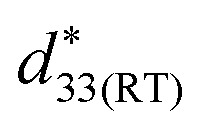
 (b). The data for PZT5H,^32^ PZT4,^10^ BNT–BT–KNN^37^ KNN–3T–0.5Mn^38^ and KNLN–BZ–BNT^39^ ceramics are taken from figures in the respective references. Temperature dependence of static *d*_33_ (c), and the planar electromechanical coupling factor (*k*_p_) normalized to its room temperature value *k*_p(RT)_ (d) for BF–0.3BT ceramics and KNN–3T ceramics.^40^

In order to further measure the temperature stability of electrical-field-induced strain, 
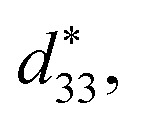
*d*_33_(*E*_0_) and *k*_p_ for BF–0.3BT ceramics, the temperature-dependent strain curves, *d*_33_(*E*_0_) and *k*_p_ from room temperature to 300 °C were tested as shown in Fig. 5. The strain under a field of 0.5 kV mm^−1^ slightly increases with increasing the temperature from 24 °C to 200 °C, and then it mildly decreases when the temperature increases from 200 °C to 300 °C as shown in Fig. 5(a). The slight increase of strain is consistent with previous results as shown in Fig. 2, which is due to the joint effect of intrinsic contribution (lattice expansion) and extrinsic contribution (domain switching). As the temperature further increases, the decrease of strain may be attributed to the increases of conductivity, which is adverse to the polarization of ceramics and dipole alignment.^30^ The temperature-dependent strain behavior (in terms of temperature-dependent 
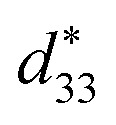
 normalized to its room temperature value 
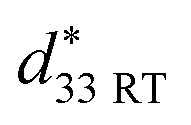
) of several representative piezoceramics is also provided in Fig. 5(b) for comparison. It is noted that the strain variation of BF–0.3BT ceramics is superior to all these piezoelectric ceramics (including PZT-5H,^32^ PZT4,^10^ KNN-3T–0.5Mn,^38^ KNLN–BZ–BNT^39^ and BNT–BT–KNN^37^). BF–0.3BT ceramics can endure temperature as high as 300 °C and the large signal 
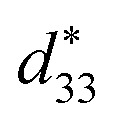
 almost has a little change from room temperature up to 300 °C. In contrast, the range of applications of the PZT-5H, PZT4, KNN-3T–0.5Mn, KNLN–BZ–BNT and BNT–BT–KNN systems is restricted below 200 °C due to their low Curie temperature *T*_C_. The temperature-dependence piezoelectric properties *d*_33_(*E*_0_) are shown in Fig. 5(c). The small signal *d*_33_(*E*_0_) also exhibit excellent temperature-insensitive behavior. This phenomenon is different from that of CZ5, in which *d*_33_ drops fast with increasing temperature in the temperature range between room temperature and 175 °C, due to the existence of polycrystalline phase boundary (PPT) around room temperature.^13^ The excellent temperature stability behavior of *d*_33_(*E*_0_) for BF–0.3BT system indicates that BF–0.3BT ceramic have a good prospects for replacing the PZT systems in the high-temperature devices. The temperature-dependence planar electromechanical coupling factor (*k*_p_), which was determined by resonance-antiresonance method at different temperatures as shown in Fig. S5,† provides the further evidence for the temperature-insensitive behavior of BF–0.3BT ceramics as shown in Fig. 5(d). The variation of *k*_p_ is less than 10% within the temperature range from room temperature up to 300 °C, which is better than textured KNN–3T ceramics.^40^ In addition, the BF–0.3BT ceramics also show excellent high-temperature anti-aging behavior, as shown in Fig. S6.† After experiencing a heat treatment at 300 °C or 400 °C for 500 h, the variation of the piezoelectric coefficient (*d*_33_) is less than 5%.

## 4. Conclusions

The temperature-dependent behaviors of ferroelectric, piezoelectric, *k*_p_ and electrical-field-induced strain were investigated to uncover the temperature stability of high-performance BF–0.3BT piezoceramics. Because of the joint effect of intrinsic (lattice expansion) and extrinsic contribution (domain switching), both electrical-field-induced strain and large signal 
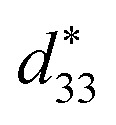
 for BF–0.3BT ceramics increased with increasing the temperature from room temperature to 180 °C. As the temperature furthering increases, the large signal *d*_33_ and electrical-field-induced strain mildly decreased, which was due to the increase of conductivity for BF–0.3BT ceramics. No matter what, the strain and 
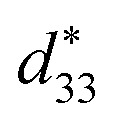
 values at 300 °C are still higher than that at room temperature. In addition, the *d*_33_(*E*_0_) and *k*_p_ exhibit excellent temperature-insensitive behavior in the wide temperature range between room temperature and 300 °C, which revealed the promising potential of using BF–0.3BT ceramics in high-temperature piezoelectric devices.

## Conflicts of interest

There are no conflicts to declare.

## Supplementary Material

RA-008-C8RA07553K-s001
