# Supplementary material for: Temperature independence of piezoelectric properties for high-performance BiFeO3–BaTiO3 lead-free piezoelectric ceramics up to 300 °C
Source: RSC Adv. 2018 Oct 19;8(62):35794–801. doi: 10.1039/c8ra07553k (PMC9088175; doi:10.1039/c8ra07553k)
Supplement: RA-008-C8RA07553K-s001 [file RA-008-C8RA07553K-s001.pdf]

Electronic Supplementary Material (ESI) for RSC Advances.
This journal is © The Royal Society of Chemistry 2018

Supplemental Materials

Temperature Independence of piezoelectric properties for High-Performance $BiFeO_3$-$BaTiO_3$ Lead-free Piezoelectric Ceramics up to 300°C

Li-Feng Zhu[1†], Qing Liu[2†], Bo-Ping Zhang[*1], Zhen-Yong Cen[2], Ke Wang[*2], Jun-jie Li[3], Yang, Bai[3], Xiao-Hui Wang [2], Jing-Feng Li[2]

[1] School of Materials Science and Engineering, University of Science and Technology Beijing, Beijing 100083, China

[2] State Key Laboratory of New Ceramics and Fine Processing, School of Materials Science and Engineering, Tsinghua University, Beijing 100084, China

[3] Key Laboratory of Environmental Fracture (Ministry of Education), University of Science and Technology Beijing, Beijing 100083, China

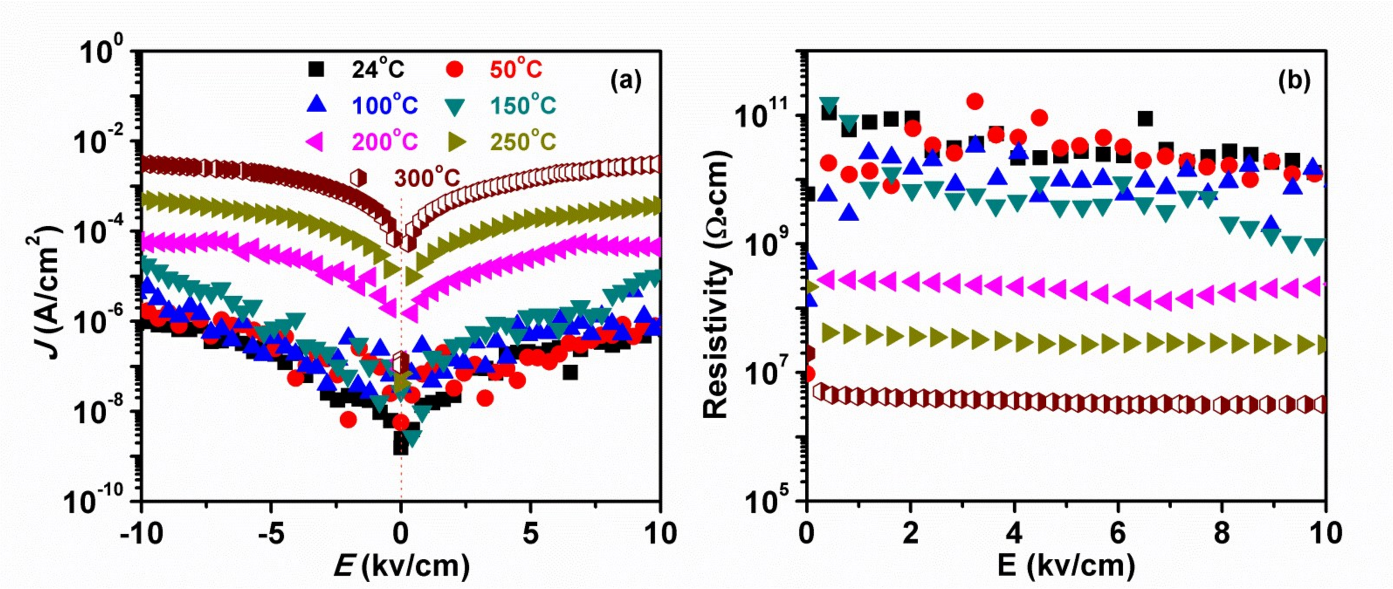

Fig.S1 Temperature dependence of leakage current (a) and resistivity (b) for BF-0.3BT ceramic

†These authors contributed equally to this work.

* Corresponding author. *E-mail address*: bpzhang@ustb.edu.cn (B.-P. Zhang), wang-ke@tsinghua.edu.cn (K. Wang)

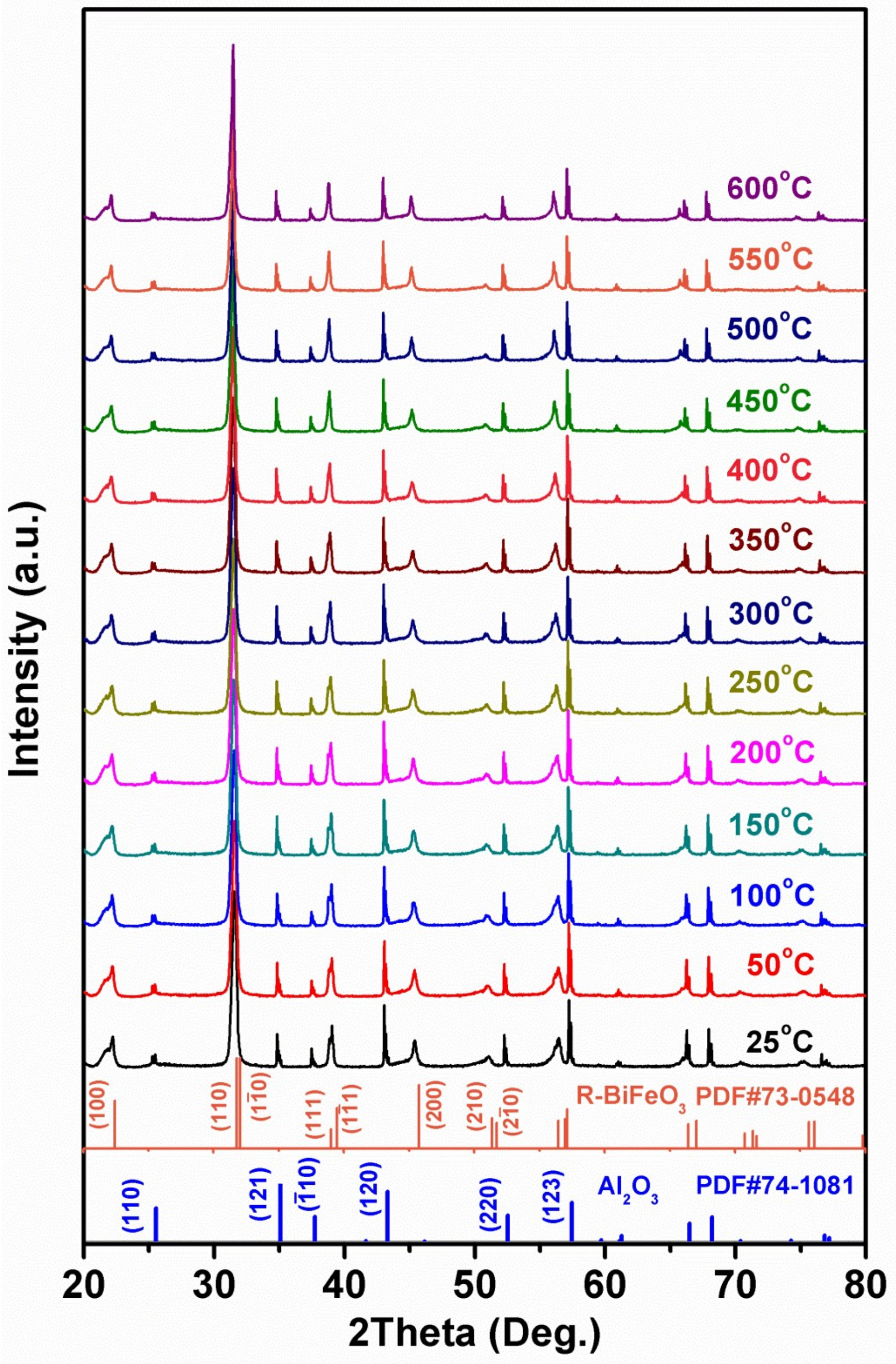

Fig S2 Temperature dependence of X-ray diffraction patterns for BF-0.3BT ceramics in a selected 2θ range of 20°-80°, the Al_2O_3 diffraction peaks are detected in all samples, which are caused by crucible.

The sample exhibit a perovskite structure without any trace of impurity phase within the detectable limit of the XRD as shown in Fig. S3. The enlarged XRD pattern in inset of Fig.S3 shows two diffraction peaks around 22°, indicating the lattice parameters are a=b≠c, which correspond to *T* phase. Thus, the phase structure of samples consists of the *R* and *T* two phases, rather than single *R* phase. This phenomenon has been verified in some papers [1, 2].

[1]. M. H. Lee, D. J. Kim, J. S. Park, S. W. Kim, T. K. Song, M. H. Kim, W. J. Kim, D. Do and I. K. Jeong, "High-Performance Lead-Free Piezoceramics with High Curie Temperatures," *Adv. Mater*. **27**, 6976-6982 (2015).

[2]. L. F. Zhu, B. P. Zhang, Z. C. Zhang, S. Li, L. J. Wang and L. J. Zheng, "Piezoelectric, ferroelectric and ferromagnetic properties of (1-x)$BiFeO_3$-x$BaTiO_3$ lead-free ceramics near morphotropic phase boundary," *J. Mater. Sci. Mater. Electron*. **29**(3)**,** 2307-2315 (2018)

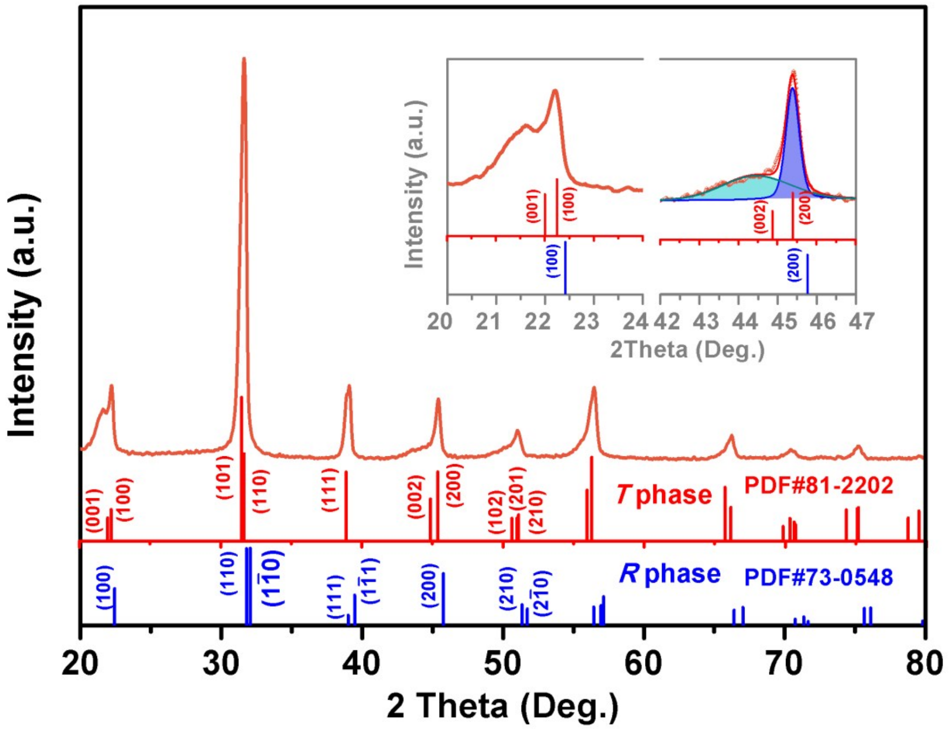

Fig. S3 X-ray diffraction patterns for BF-0.3BT ceramics in a selected 2θ range of 20°-80° measured at room temperature. The standard diffraction peaks cited from $BiFeO_3$ (BF) with *R* symmetry (PDF#73-0548) and $BaTiO_3$ (BT) with *T* phase (PDF#81-2202) are indicated by vertical lines for comparison.

Fig.S4 shows minor strain-field hysteresis loops were used to calculate the complex and imaginary piezoelectric coefficients. Following the equations (S1-S3), the real part of d_{33} (d') was calculated. In addition, the extrinsic contribution ratio for BF-0.3BT ceramics was also evaluated as following the equation (S4 and S5).

$$d'' = A_{EM} / \pi E_0^2 \qquad (S1)$$

$$d^* = \chi_{p-p} / 2E_0 \qquad (S2)$$

$$d' = \sqrt{d^{*2} - d''^2} \qquad (S3)$$

$$d' = d_{init} + a_d E_0 \qquad (S4)$$

$$Extrinsic(\%) = d_{extrinsic} \Big/ (d_{instrinsic} + d_{extrinsic}) \times 100\% = a_d E_0 \Big/ (d_{init} + a_d E_0) \times 100\% \qquad (S5)$$

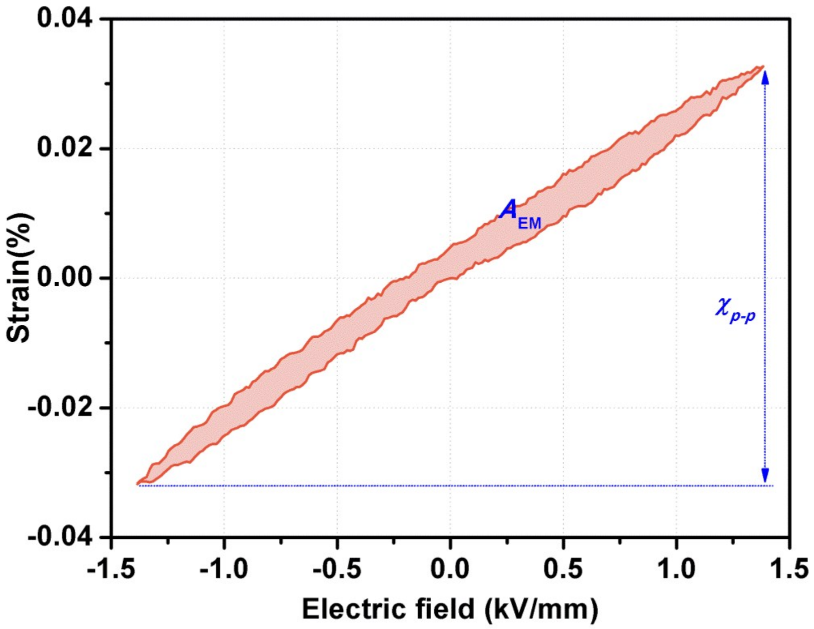

Fig. S4 (Color online) Minor strain-field hysteresis loops were used to calculate the complex and imaginary piezoelectric coefficients. A typical minor hysteresis loop is presented (BF-0.3BT), measured at 5 Hz and 1.4kV/cm

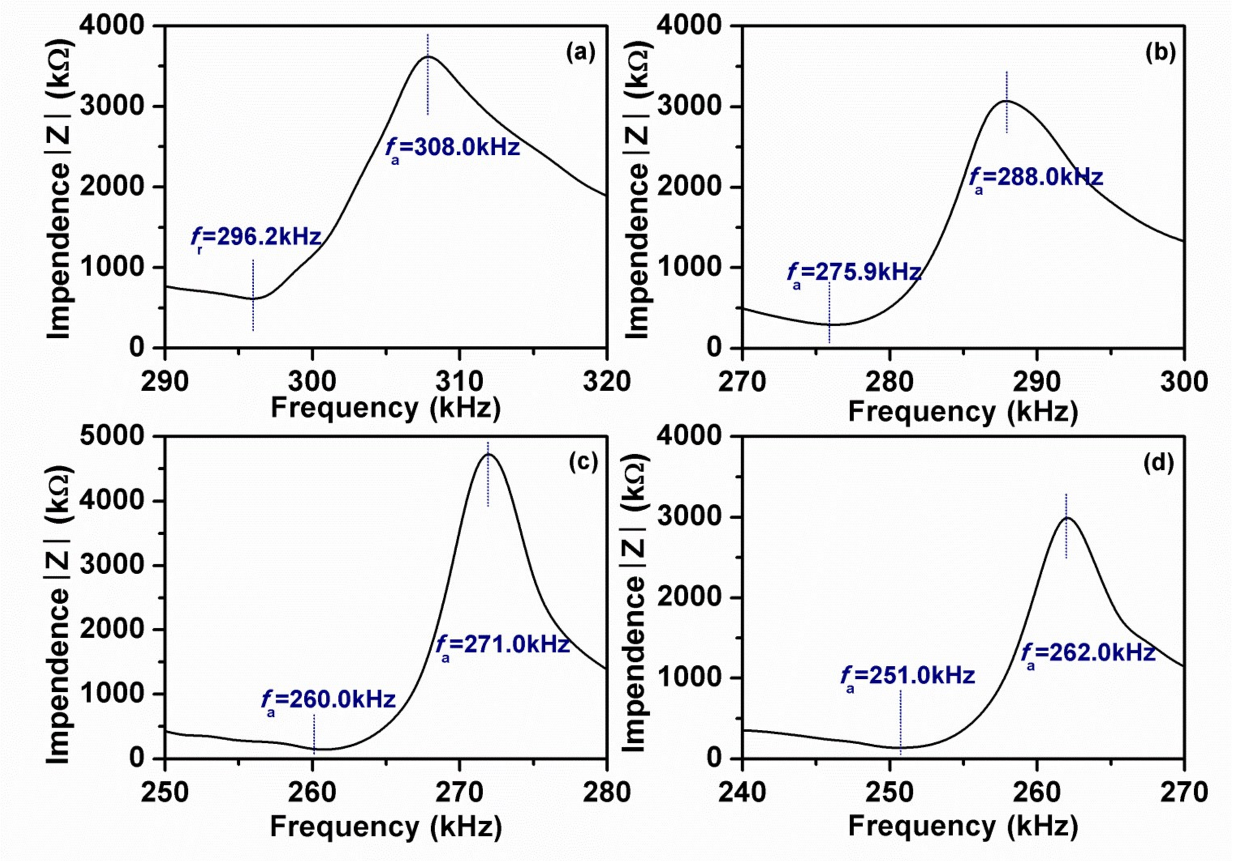

Fig. S5 The impedance spectra |Z| as a function of frequency of BF-0.3BT ceramics with different measured temperature, (a) 30 ^{o}C, (b) 100 ^{o}C, (c) 200 ^{o}C and (d) 300 ^{o}C

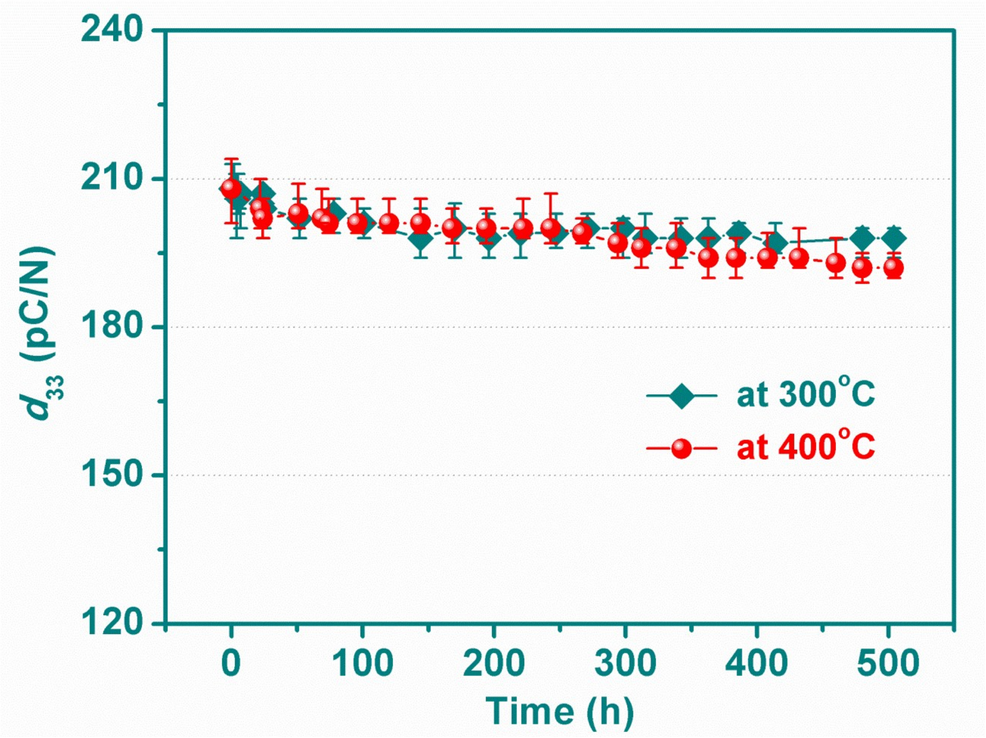

Fig. S6 Aging of piezoelectric coefficient (d_{33}) of BF-0.3BT ceramics was measured after heat treatment at 300 ^{o}C and 400 ^{o}C
